# Perinatal neuroprotection update

**DOI:** 10.12688/f1000research.8546.1

**Published:** 2016-08-09

**Authors:** Angie C. Jelin, Kirsten Salmeen, Dawn Gano, Irina Burd, Mari-Paule Thiet

**Affiliations:** 1 Department of Gynecology and Obstetrics, Johns Hopkins Hospital, Baltimore, MD, USA; 2 Department of Obstetrics, Gynecology and Reproductive Sciences, University of California San Francisco, San Francisco, CA, USA; 3 Departments of Neurology & Pediatrics, University of California San Francisco, San Francisco, CA, USA

**Keywords:** neuroprotection, Perinatal, neurological deficits, Delayed umbilical cord clamping, preterm delivery, Zika virus

## Abstract

Antepartum, intrapartum, and neonatal events can result in a spectrum of long-term neurological sequelae, including cerebral palsy, cognitive delay, schizophrenia, and autism spectrum disorders [1]. Advances in obstetrical and neonatal care have led to survival at earlier gestational ages and consequently increasing numbers of periviable infants who are at significant risk for long-term neurological deficits. Therefore, efforts to decrease and prevent cerebral insults attempt not only to decrease preterm delivery but also to improve neurological outcomes in infants delivered preterm. We recently published a comprehensive review addressing the impacts of magnesium sulfate, therapeutic hypothermia, delayed cord clamping, infections, and prevention of preterm delivery on the modification of neurological risk [2]. In this review, we will briefly provide updates to the aforementioned topics as well as an expansion on avoidance of toxin and infections, specifically the Zika virus.

## Introduction

Neurological insults result in significant immediate and long-term physical, emotional, and financial costs. Perinatal events can lead to long-term outcomes, including cerebral palsy (CP) and autism spectrum disorders. Clinicians desire to minimize adverse outcomes and maximize neuroprotective options. In recent years, the research in this area has been extensive and has included successes, failures, and inconclusive results. Owing to the spectrum and variation in long-term outcomes, most research has focused on a defined neurological entity such as CP or autism spectrum disorder. CP is used to describe a clinical spectrum of neurological impairment that may arise from various etiologies ^3^. The majority of cases are congenital. CP occurs in 2 per 1,000 infants but is inversely affected by early gestational age and low birth weight, affecting 60 per 1,000 infants that weigh less than 1,500 g ^4^. It is speculated that a decrease in preterm delivery would significantly reduce the incidence of CP, leading to the impetus for research on preterm birth preventive strategies such as progesterone.

At this time, there is support for many different management strategies that have the potential to minimize adverse neurological outcomes. The most widely used strategies are avoidance and prevention of risk factors such as infections, including the Zika virus, and progesterone to prevent preterm delivery. Magnesium sulfate and delayed cord clamping (DCC) have been accepted as prophylactic strategies. Treatment with therapeutic hypothermia in the neonatal period minimizes the impact of a neurological insult after it occurs.

## Antepartum

### Prevention of preterm delivery

The risks of intraventricular hemorrhage (IVH), CP, and neurological impairment are closely related to gestational age of delivery ^5, 6^; thus, prevention of preterm delivery is perhaps the most effective strategy for neonatal neuroprotection. There are currently three preventative options to consider: progesterone, cerclage, and pessary.

Although the functional properties of progesterone are not fully understood, a number of studies have demonstrated the efficacy of progesterone in the prevention of preterm birth for women with a history of preterm birth or a short cervix visualized on ultrasound ^7^. In 2012, the American College of Obstetricians and Gynecologists (ACOG) recommended the use of weekly intramuscular hydroxyprogesterone caproate starting at 16 to 24 weeks’ gestation for women with a singleton pregnancy and a history of singleton preterm delivery ^8^. This intervention has been demonstrated to reduce the risk of recurrent preterm birth by 40% ^7^.

Vaginal progesterone is preferred for women who do not have a history of preterm delivery but who have a cervical length of less than 20 mm on ultrasound. This practice is supported by a meta-analysis of five randomized controlled trials (RCTs), which demonstrate a reduction in preterm birth of less than 33 weeks in the vaginal progesterone cohort (12.4% versus 22.0%; relative risk [RR] 0.58, 95% confidence interval [CI] 0.42–0.08) ^9^.

The use of cervical cerclage is indicated among women with a history of preterm birth who are found to have a cervical length of less than 2.5 cm on ultrasound. Otherwise, cerclage is not recommended in the absence of a prior preterm delivery ^6^.

Use of a cervical pessary has been examined extensively. In a study of 385 singleton pregnancies with a short cervix, a pessary was associated with a decrease in preterm deliveries of less than 34 weeks (6% versus 27%; odds ratio [OR] 0.18, 95% CI 0.08–0.37) ^10^. Several subsequent studies, however, failed to duplicate these results ^11– 13^. One subsequent study, by Fox *et al*., reported fewer deliveries prior to 32 weeks’ gestation with the addition of a cervical pessary to vaginal progesterone in twins with a short cervix (4.8% versus 28.6%, *P* = 0.05) ^14^.

In summary, weekly intramuscular hydroxyprogesterone caproate is recommended to reduce the risk of recurrent preterm birth among women with singleton pregnancies and a history of preterm birth, and vaginal progesterone is recommended for women with singleton pregnancies and a short cervix identified by ultrasound. Cerclage may be considered for women with a history of preterm birth who have cervical shortening despite weekly hydroxyprogesterone caproate. Pessary is unlikely to cause harm, but evidence to support its use remains mixed.

### Magnesium sulfate

The neuroprotective effects of magnesium sulfate were documented in 1987 ^15^ because of the observation of a serendipitous decrease in IVH in infants delivered to women with preeclampsia. Results were replicated by Kuban *et al*. in 1992 with IVH rates of 4.4% versus 18.9% in infants delivered to those who received magnesium sulfate versus those who did not ^16^. These observational studies were supported by five RCTs ^17– 21^.

The proposed benefits of magnesium sulfate were accepted by the ACOG, and intrapartum magnesium is the standard of care for women at less than 32 weeks’ gestation who are at risk for delivery within 7 days ^22^. Although studies have attempted to solidify the optimal dose of administration, a meta-analysis of three RCTs including 360 women failed to demonstrate superiority of a specific dosing strategy with analysis of neonatal or maternal morbidity ^23^. We therefore suggest that any combination of a 4 to 6 g load followed by 1 to 2 g/hour continuous infusion can be used; however, safety experts advocate that labor-and-delivery units use a single, standardized protocol to reduce the risk of medication ^22^.

Meta-analyses have shown that antenatal magnesium sulfate for neuroprotection in preterm infants is associated with a reduction of CP at a corrected age of 18 to 24 months ^24– 26^.

Long-term childhood studies have not demonstrated improved functionality in exposed newborns. A French RCT with an assessment of 503 children found a trend toward benefits in death/motor dysfunction (OR 0.79, 95% CI 0.53–1.17) or cognitive difficulties (OR 0.89, 95% CI 0.59–1.33) in school-age children after *in utero* exposure to magnesium sulfate, but the benefits were not significant ^27^. In a trial of 1,255 infants, Doyle *et al*. evaluated school-age children and demonstrated no differences in CP (OR 1.26, 95% CI 0.84–1.91) or abnormal motor function (OR 1.16, 95% CI 0.88–1.52) ^28^. In addition, there is some concern about the associated risk of neonatal mortality (RR 0.8, 95% CI 0.62–1.03).

The mechanism underlying the neuroprotective effects of magnesium sulfate is not known. Proposed mechanisms include stabilization of rapid fluctuations in blood pressure, increased cerebral blood flow, and decreasing inflammation after hypoxia-ischemia and calcium-induced excitotoxicity ^29– 32^. However, antenatal magnesium sulfate was not associated with a decreased risk of brain injuries known to cause CP, such as severe IVH or cystic white matter injury, in several randomized trials that used ultrasound to diagnose brain injury ^24– 28^. A subset analysis of one randomized trial ^28^ focused on newborns less than 32 weeks at birth and showed that magnesium sulfate was associated with a reduced risk of echolucencies and echodensities on cranial ultrasound ^33^. A recent study has also shown that antenatal magnesium sulfate is associated with a reduction of cerebellar hemorrhage on magnetic resonance imaging (MRI) obtained soon after birth in preterm newborns ^34^.

### Zika

Infections, including chorioamnionitis, cytomegalovirus, rubella, varicella, toxoplasmosis, and now Zika virus, have been associated with neurological impairment in the fetus. Concern about the neurological effects of viral infections has been increased by Zika virus awareness because of a recent increase in neurological abnormalities of infants in Brazil ^35^.

Zika is a *Flaviviridae* virus that is transmitted by *Aedes* species mosquitoes. Initial cases of vertical transmission of Zika virus were recently described in French Polynesia in 2013 ^36^, followed by an outbreak of Zika in Brazil in May 2015 ^1^ that raised concern due to its association with neonatal microcephaly. The initial reports of causation were speculative ^37^, but pathological studies have strengthened the association, which is now generally accepted as a major public health concern ^38, 39^. Vertical transmission from mother to fetus appears to be responsible for many cases of fetal brain abnormalities and microcephaly ^40^. Transmission of the Zika virus has been reported in all three trimesters; however, more severe transmission is suggested to occur in the first trimester ^41^. There is now biological plausibility based on confirmation of Zika by reverse transcriptase-polymerase chain reaction assay in affected fetal brain tissue ^42^. This new outbreak has reminded us of vertical transmission risks of other infections in the Flaviviridae family, including hepatitis C (in humans) and bovine viral diarrhea virus (in cattle), known to cause hydrocephalus and microcephaly in young ^43^.

In the absence of vaccines and treatments, for which research is in progress ^44^, the current recommendations for women who are pregnant or immediately pre-conception are to avoid travel to regions that are affected by Zika virus and for women residing in affected regions to avoid pregnancy altogether. Most affected are asymptomatic ^45^, and so for those exposed, testing algorithms were developed with recommendations for serial fetal ultrasounds if testing is positive or fetal abnormalities are identified. There have been multiple guidelines ^46, 47^ and Centers for Disease Control and Prevention updates ^46, 48– 50^; the most recent, published in March 2016, reported 39 affected countries or territories and recommended that women who have symptoms wait at least 8 weeks after onset to attempt conception and men wait 6 months. Men or women with possible exposure should wait 8 weeks ^51^.

Although the Zika virus is newly identified, the potential for infectious diseases to cause harm to the developing fetal brain has been well documented. Ideally, pregnant women would have the opportunity to know their risk of infection and status of immunity and be equipped with strategies to avoid infection. The development of genetically based testing algorithms that search widely for viral or bacterial genetic material (or both) hold promise to help with diagnosis, but without treatment or vaccines, the only option for pregnant women is avoidance of exposure.

## Intrapartum

### Delayed umbilical cord clamping

DCC has been associated with a potential 50% reduction in the risk of IVH in preterm infants, and therefore in 2012 the American Academy of Pediatrics and ACOG issued a committee opinion recommending a more-than-30-second delay in cord clamping for preterm infants ^51^. This opinion was supported by several RCTs demonstrating increases in neonatal hematocrit (with reductions in the need for blood transfusion), reduced need for volume resuscitation, and decreases in IVH but without an increase in the risk of hyperbilirubinemia or other complications ^52– 55^. Proposed mechanisms for the benefits associated with DCC include an improved cardiovascular transition with ventilation prior to umbilical cord clamping ^53, 56^.

The optimal timing for cord clamping in preterm infants is not known. A recent prospective cohort study of infants at less than 32 weeks comparing DCC 30–45 seconds versus 60–75 seconds suggested that longer delay was associated with reductions in hypothermia on admission (1% versus 5%, *P* = 0.01), surfactant therapy (13% versus 28%, *P* = 0.001), any intubation (27% versus 40%, *P* = 0.007), and any red blood cell transfusion (20% versus 33%, *P* = 0.008) during the hospitalization ^57^. Umbilical cord milking may be an alternative to DCC; one study showed improved outcomes with milking over DCC ^58^, although a long-term follow-up study showed no difference in Bayley III scores with umbilical cord milking as compared with DCC ^59^. Other studies have been somewhat less clear with regard to the benefits of DCC. An RCT of 200 patients at less than 34 weeks’ gestation showed no difference in rates of transfusion despite a statistically significant difference in initial hemoglobin ^56^. In that study, the rate of IVH was not statistically significantly different, although it tended toward a difference with 11.1% of delayed-clamping infants experiencing IVH versus 19.8% in the control group ( *P* = 0.09). Another RCT, which included long-term follow-up of 208 preterm deliveries assessed at 18 to 22 months, showed no initial difference in IVH; however, Bayley scale motor scores were less likely to be below 85 (OR 0.32, 95% CI 0.10–0.90, *P* = 0.03) in the DCC group ^55^.

For full-term infants, in whom the risk of IVH, hypotension, and need for transfusion is drastically lower, the potential benefits of DCC are primarily related to long-term anemia and its associated impacts on neurological development. In a recent meta-analysis of 15 studies with a total of 3,911 term gestations (>37 weeks), infants with immediate cord clamping were more than twice as likely to be iron-deficient at 3 to 6 months compared with DCC (RR 2.65, 95% CI 1.04–6.73). There were no differences in maternal outcomes, specifically in postpartum hemorrhage (RR 1.17, 95% CI 0.94–1.44); however, fewer infants in the immediate cord clamp group required phototherapy for jaundice than in the DCC group (RR 0.62, 95% CI 0.41–0.96) ^60^.

In summary, although the data supporting the use of DCC are somewhat mixed, adequate studies demonstrate a benefit, particularly among preterm infants, and there is no evidence for significant harm. Thus, as a strategy for neuroprotection, a delay in umbilical cord clamping of at least 30 seconds is recommended.

## Neonatal

### Therapeutic hypothermia

Therapeutic hypothermia is the standard of care for the treatment of hypoxic-ischemic encephalopathy (HIE) in term newborns ^61, 62^. Experiments in newborn animal models demonstrated that hypothermia initiated within 5.5 hours following a hypoxic-ischemic insult resulted in improved neuropathological and functional outcomes ^63, 64^. After the benefit of hypothermia in animal models was shown, clinical studies in term newborns were pursued to establish feasibility and safety ^65, 66^. Multiple large RCTs of therapeutic hypothermia in term newborns were subsequently pursued and this demonstrated that both selective head cooling and whole-body cooling are effective treatments for HIE ^67– 72^. However, whole-body hypothermia has the advantage of leaving the scalp accessible for electrophysiologic monitoring with electroencephalography (EEG) or amplitude-integrated EEG or both. Adverse effects of hypothermia include sinus bradycardia, lipolysis, and electrolyte abnormalities ^75^.

Therapeutic hypothermia is associated with a reduction of seizures, and brain injury on MRI, as well as improved neurodevelopmental outcomes ^73– 75^. Advanced MRI studies have also shown that hypothermia is associated with improved markers of brain microstructure and metabolism ^76, 77^. Meta-analysis of the 11 RCTs, which included 1,505 term newborns in total, indicated that hypothermia is associated with a significant reduction of death or neurodevelopmental disability at 18 months (risk ratio 0.75, 95% CI 0.68–0.83; number needed to treat 7, 95% CI 5–10) ^72^. Long-term follow-up of treated newborns has illustrated that the benefits of therapeutic hypothermia are sustained through middle childhood up to 7 years ^78– 80^.

Current clinical recommendations are to initiate hypothermia within 6 hours of birth in newborns older than 36 weeks at birth with moderate to severe encephalopathy and to continue hypothermia for 72 hours. Therapeutic hypothermia requires considerable resources and specialized equipment that are typically available only in tertiary care centers. Disseminating a broad community awareness of the symptoms of HIE and of the importance of prompt identification of affected newborns to enable initiation of hypothermia is critical to optimize access to this effective neuroprotective therapy.

Although neurodevelopmental outcomes after neonatal HIE have improved since the advent of therapeutic hypothermia, disability and executive dysfunction remain common, highlighting the urgent need for additional neuroprotective strategies. One such strategy is the optimization of therapeutic hypothermia. Shankaran *et al*. conducted a randomized trial to determine whether longer duration of cooling (120 hours) or deeper degree of cooling (32°C) or both are superior to cooling to 33.5°C for 72 hours ^81^. The trial was closed early after a futility analysis revealed that longer cooling or deeper cooling or both did not reduce neonatal death. There are ongoing RCTs evaluating hypothermia initiated between 6 and 24 hours of age and continued for 96 hours in term newborns at least 36 weeks at birth (NCT00614744) as well as the safety and effectiveness of hypothermia for 72 hours in preterm infants with a gestational age of 33 to 35 weeks who present at less than 6 hours with moderate to severe neonatal encephalopathy (NCT01793129).

### Adjunctive therapies for hypoxic-ischemic encephalopathy

A number of promising neuroprotective agents are being evaluated as adjunctive therapies to therapeutic hypothermia. The ultimate goal is to identify subsets of patients who would benefit from a particular “cocktail” of adjunctive agents plus hypothermia in order to improve long-term outcomes after HIE. A strong body of animal model evidence supports the neuroprotective effects of erythropoietin ^82, 83^. In a phase I trial of erythropoietin plus hypothermia, Wu *et al*. demonstrated safety and feasibility of erythropoietin as an add-on therapy ^84^. Wu *et al*. have recently completed a phase II RCT of erythropoietin plus hypothermia in 50 newborns with moderate to severe HIE and showed that erythropoietin-treated newborns had lower global brain injury scores on MRI after rewarming as well as improved motor function at 1 year ^85^.

Xenon had abundant animal model evidence of a strong neuroprotective effect in combination with hypothermia; however, a proof-of-concept trial of hypothermia plus xenon in term newborns was recently stopped early because of a lack of benefit ^86^. Trials of topiramate (NCT 01241019 and NCT 01765218), melatonin (NCT02621944), and clonidine (NCT 01862250) concurrent to hypothermia are under way. There continues to be interest in the use of banked umbilical cord blood for the treatment of HIE ^83^, and a trial that planned to evaluate the safety and effectiveness of cord blood- and placenta-derived stem cells in term infants with severe HIE has yet to begin recruitment (NCT02434965). In addition, stem cells are being investigated for the treatment of CP resulting from heterogeneous etiologies, including neonatal HIE. Short-term follow-up in 328 participants with CP from five trials has indicated a small statistically significant beneficial effect of stem cell treatment on gross motor skills ^87^.

## Future directions

We have summarized the recent literature on perinatal neuroprotection; however, despite advances in prevention and treatment, the incidence in long-term adverse outcomes, such as autism, appears to be rising. Preterm delivery is far from being eliminated, and infants are surviving at younger gestational age. Vaccinations and treatments for viruses seem poised to effectively treat Zika, but additional viruses and infections will likely emerge. Management strategies to reduce the risk of neurological insult in preterm and term infants are imperative. In addition, effective treatment in adjunct to therapeutic hyperthermia is desirable.

## Abbreviations

ACOG, American College of Obstetricians and Gynecologists; CI, confidence interval; CP, cerebral palsy; DCC, delayed cord clamping; EEG, electroencephalography; HIE, hypoxic-ischemic encephalopathy; IVH, intraventricular hemorrhage; MRI, magnetic resonance imaging; OR, odds ratio; RCT, randomized controlled trial; RR, relative risk.

## References

[1] Padilla N Eklöf E Mårtensson GE: Poor Brain Growth in Extremely Preterm Neonates Long Before the Onset of Autism Spectrum Disorder Symptoms. *Cereb Cortex.* 2015; pii: bhv300. 10.1093/cercor/bhv300 26689588

[2] Salmeen KE Jelin AC Thiet MP: Perinatal neuroprotection. *F1000Prime Rep.* 2014;6:6. 10.12703/P6-6 24592318 PMC3883423

[3] Rosenbaum P Paneth N Leviton A: A report: the definition and classification of cerebral palsy April 2006. *Dev Med Child Neurol Suppl.* 2007;109:8–14. 17370477

[4] Winter S Autry A Boyle C: Trends in the prevalence of cerebral palsy in a population-based study. *Pediatrics.* 2002;110(6):1220–5. 12456922 10.1542/peds.110.6.1220

[5] Stoll BJ Hansen NI Bell EF: Neonatal outcomes of extremely preterm infants from the NICHD Neonatal Research Network. *Pediatrics.* 2010;126(3):443–56. 10.1542/peds.2009-2959 20732945 PMC2982806

[6] Moore T Hennessy EM Myles J: Neurological and developmental outcome in extremely preterm children born in England in 1995 and 2006: the EPICure studies. *BMJ.* 2012;345:e7961. 10.1136/bmj.e7961 23212880 PMC3514471

[7] Meis PJ Klebanoff M Thom E: Prevention of recurrent preterm delivery by 17 alpha-hydroxyprogesterone caproate. *N Engl J Med.* 2003;348(24):2379–85. 10.1056/NEJMoa035140 12802023

[8] Committee on Practice Bulletins—Obstetrics, The American College of Obstetricians and Gynecologists: Practice bulletin no. 130: prediction and prevention of preterm birth. *Obstet Gynecol.* 2012;120(4):964–73. 10.1097/AOG.0b013e3182723b1b 22996126

[9] Romero R Nicolaides K Conde-Agudelo A: Vaginal progesterone in women with an asymptomatic sonographic short cervix in the midtrimester decreases preterm delivery and neonatal morbidity: a systematic review and metaanalysis of individual patient data. *Am J Obstet Gynecol.* 2012;206(2):124.e1–19. 10.1016/j.ajog.2011.12.003 22284156 PMC3437773

[10] Abdel-Aleem H Shaaban OM Abdel-Aleem MA: Cervical pessary for preventing preterm birth. *Cochrane Database Syst Rev.* 2013; (5):CD007873. 10.1002/14651858.CD007873.pub3 23728668 PMC6491132

[11] Hui SY Chor CM Lau TK: Cerclage pessary for preventing preterm birth in women with a singleton pregnancy and a short cervix at 20 to 24 weeks: a randomized controlled trial. *Am J Perinatol.* 2013;30(4):283–8. 10.1055/s-0032-1322550 22875662

[12] Nicolaides KH Syngelaki A Poon LC: A Randomized Trial of a Cervical Pessary to Prevent Preterm Singleton Birth. *N Engl J Med.* 2016;374(11):1044–52. 10.1056/NEJMoa1511014 26981934

[13] Stricker N Timmesfeld N Kyvernitakis I: Vaginal progesterone combined with cervical pessary: A chance for pregnancies at risk for preterm birth? *Am J Obstet Gynecol.* 2016;214(6):739.e1–739.e10. 10.1016/j.ajog.2015.12.007 26692180

[14] Fox NS Gupta S Lam-Rachlin J: Cervical Pessary and Vaginal Progesterone in Twin Pregnancies With a Short Cervix. *Obstet Gynecol.* 2016;127(4):625–30. 10.1097/AOG.0000000000001300 26959202

[15] van de Bor M Verloove-Vanhorick SP Brand R: Incidence and prediction of periventricular-intraventricular hemorrhage in very preterm infants. *J Perinat Med.* 1987;15(4):333–9. 10.1515/jpme.1987.15.4.333 3325633

[16] Kuban KC Leviton A Pagano M: Maternal toxemia is associated with reduced incidence of germinal matrix hemorrhage in premature babies. *J Child Neurol.* 1992;7(1):70–6. 10.1177/088307389200700113 1552156

[17] Mittendorf R Dambrosia J Pryde PG: Association between the use of antenatal magnesium sulfate in preterm labor and adverse health outcomes in infants. *Am J Obstet Gynecol.* 2002;186(6):1111–8. 10.1067/mob.2002.123544 12066082

[18] Crowther CA Hiller JE Doyle LW: Effect of magnesium sulfate given for neuroprotection before preterm birth: a randomized controlled trial. *JAMA.* 2003;290(20):2669–76. 10.1001/jama.290.20.2669 14645308

[19] Magpie Trial Follow-Up Study Collaborative Group: The Magpie Trial: a randomised trial comparing magnesium sulphate with placebo for pre-eclampsia. Outcome for children at 18 months. *BJOG.* 2007;114(3):289–99. 10.1111/j.1471-0528.2006.01165.x 17166221 PMC2063969

[20] Marret S Marpeau L Zupan-Simunek V: Magnesium sulphate given before very-preterm birth to protect infant brain: the randomised controlled PREMAG trial*. *BJOG.* 2007;114(3):310–8. 10.1111/j.1471-0528.2006.01162.x 17169012

[21] Rouse DJ Hirtz DG Thom E: A randomized, controlled trial of magnesium sulfate for the prevention of cerebral palsy. *N Engl J Med.* 2008;359(9):895–905. 10.1056/NEJMoa0801187 18753646 PMC2803083

[22] Committee Opinion No 652: Magnesium Sulfate Use in Obstetrics. *Obstet Gynecol.* 2016;127(1):e52–3. 10.1097/AOG.0000000000001267 26695587

[23] Bain E Middleton P Crowther CA: Different magnesium sulphate regimens for neuroprotection of the fetus for women at risk of preterm birth. *Cochrane Database Syst Rev.* 2012; (2):CD009302. 10.1002/14651858.CD009302.pub2 22336863 PMC11472847

[24] Conde-Agudelo A Romero R: Antenatal magnesium sulfate for the prevention of cerebral palsy in preterm infants less than 34 weeks' gestation: a systematic review and metaanalysis. *Am J Obstet Gynecol.* 2009;200(6):595–609. 10.1016/j.ajog.2009.04.005 19482113 PMC3459676

[25] Costantine MM Weiner SJ Eunice Kennedy Shriver National Institute of Child Health and Human Development Maternal-Fetal Medicine Units Network: Effects of antenatal exposure to magnesium sulfate on neuroprotection and mortality in preterm infants: a meta-analysis. *Obstet Gynecol.* 2009;114(2 Pt 1):354–64. 10.1097/AOG.0b013e3181ae98c2 19622997 PMC2761069

[26] Doyle LW Crowther CA Middleton P: Magnesium sulphate for women at risk of preterm birth for neuroprotection of the fetus. *Cochrane Database Syst Rev.* 2009; (1):CD004661. 10.1002/14651858.CD004661.pub3 17636771

[27] Chollat C Enser M Houivet E: School-age outcomes following a randomized controlled trial of magnesium sulfate for neuroprotection of preterm infants. *J Pediatr.* 2014;165(2):398–400.e3. 10.1016/j.jpeds.2014.04.007 24837863

[28] Doyle LW Anderson PJ Haslam R: School-age outcomes of very preterm infants after antenatal treatment with magnesium sulfate vs placebo. *JAMA.* 2014;312(11):1105–13. 10.1001/jama.2014.11189 25226476

[29] Nelson KB Grether JK: Causes of cerebral palsy. *Curr Opin Pediatr.* 1999;11(6):487–91. 10590904 10.1097/00008480-199912000-00002

[30] Burd I Breen K Friedman A: Magnesium sulfate reduces inflammation-associated brain injury in fetal mice. *Am J Obstet Gynecol.* 2010;202(3):292.e1–9. 10.1016/j.ajog.2010.01.022 20207246 PMC2835629

[31] McDonald JW Silverstein FS Johnston MV: Magnesium reduces *N*-methyl-D-aspartate (NMDA)-mediated brain injury in perinatal rats. *Neurosci Lett.* 1990;109(1–2):234–8. 10.1016/0304-3940(90)90569-U 2179770

[32] Galinsky R Davidson JO Drury PP: Magnesium sulphate and cardiovascular and cerebrovascular adaptations to asphyxia in preterm fetal sheep. *J Physiol.* 2016;594(5):1281–93. 10.1113/JP270614 26077461 PMC4771788

[33] Hirtz DG Weiner SJ Bulas D: Antenatal Magnesium and Cerebral Palsy in Preterm Infants. *J Pediatr.* 2015;167(4):834–839.e3. 10.1016/j.jpeds.2015.06.067 26254839 PMC4587284

[34] Gano D Ho ML Partridge JC: Antenatal Exposure to Magnesium Sulfate Is Associated with Reduced Cerebellar Hemorrhage in Preterm Newborns. *J Pediatr.* 2016; pii: S0022-3476(16)30482-6. In Press. 10.1016/j.jpeds.2016.06.053 27453378 PMC5085851

[35] Schuler-Faccini L Ribeiro EM Feitosa IM: Possible Association Between Zika Virus Infection and Microcephaly - Brazil, 2015. *MMWR Morb Mortal Wkly Rep.* 2016;65(3):59–62. 10.15585/mmwr.mm6503e2 26820244

[36] Besnard M Lastere S Teissier A: Evidence of perinatal transmission of Zika virus, French Polynesia, December 2013 and February 2014. *Euro Surveill.* 2014;19(13): pii: 20751. 10.2807/1560-7917.ES2014.19.13.20751 24721538

[37] Butler D: Zika virus: Brazil's surge in small-headed babies questioned by report. *Nature.* 2016;530(7588):13–4. 10.1038/nature.2016.19259 26842033

[38] Burd I Griffin D: The chasm between public health and reproductive research: what history tells us about Zika virus. *J Assist Reprod Genet.* 2016;33(4):439–40. 10.1007/s10815-016-0687-3 26943916 PMC4818626

[39] Dyer O: Zika virus spreads across Americas as concerns mount over birth defects. *BMJ.* 2015;351:h6983. 10.1136/bmj.h6983 26698165

[40] Brasil P Pereira JP Jr Raja Gabaglia C: Zika Virus Infection in Pregnant Women in Rio de Janeiro - Preliminary Report. *N Engl J Med.* 2016. 10.1056/NEJMoa1602412 26943629 PMC5323261

[41] Cauchemez S Besnard M Bompard P: Association between Zika virus and microcephaly in French Polynesia, 2013-15: a retrospective study. *Lancet.* 2016;387(10033):2125–32. 10.1016/S0140-6736(16)00651-6 26993883 PMC4909533

[42] Mlakar J Korva M Tul N: Zika Virus Associated with Microcephaly. *N Engl J Med.* 2016;374(10):951–8. 10.1056/NEJMoa1600651 26862926

[43] Badman RT Mitchell G Jones RT: Association of bovine viral diarrhoea virus infection to hydranencephaly and other central nervous system lesions in perinatal calves. *Aust Vet J.* 1981;57(6):306–7. 10.1111/j.1751-0813.1981.tb05831.x 7316899

[44] Palacios R Poland GA Kalil J: Another emerging arbovirus, another emerging vaccine: Targeting Zika virus. *Vaccine.* 2016;34(20):2291–3. 10.1016/j.vaccine.2016.03.059 27015734

[45] Duffy MR Chen TH Hancock WT: Zika virus outbreak on Yap Island, Federated States of Micronesia. *N Engl J Med.* 2009;360(24):2536–43. 10.1056/NEJMoa0805715 19516034

[46] Petersen EE Staples JE Meaney-Delman D: Interim Guidelines for Pregnant Women During a Zika Virus Outbreak--United States, 2016. *MMWR Morb Mortal Wkly Rep.* 2016;65(2):30–3. 10.15585/mmwr.mm6502e1 26796813

[47] Meaney-Delman D Rasmussen SA Staples JE: Zika Virus and Pregnancy: What Obstetric Health Care Providers Need to Know. *Obstet Gynecol.* 2016;127(4):642–8. 10.1097/AOG.0000000000001378 26889662

[48] Fleming-Dutra KE Nelson JM Fischer M: Update: Interim Guidelines for Health Care Providers Caring for Infants and Children with Possible Zika Virus Infection--United States, February 2016. *MMWR Morb Mortal Wkly Rep.* 2016;65(7):182–7. 10.15585/mmwr.mm6507e1 26914500

[49] Vouga M Musso D van Mieghem T: CDC guidelines for pregnant women during the Zika virus outbreak. *Lancet.* 2016;387(10021):843–4. 10.1016/S0140-6736(16)00383-4 26898855

[50] Petersen EE Polen KN Meaney-Delman D: Update: Interim Guidance for Health Care Providers Caring for Women of Reproductive Age with Possible Zika Virus Exposure - United States, 2016. *MMWR Morb Mortal Wkly Rep.* 2016;65(12):315–22. 10.15585/mmwr.mm6512e2 27031943

[51] Committee on Obstetric Practice, American College of Obstetricians and Gynecologists: Committee Opinion No.543: Timing of umbilical cord clamping after birth. *Obstet Gynecol.* 2012;120(6):1522–6. 10.1097/01.AOG.0000423817.47165.48 23168790

[52] Ruangkit C Moroney V Viswanathan S: Safety and efficacy of delayed umbilical cord clamping in multiple and singleton premature infants - A quality improvement study. *J Neonatal Perinatal Med.* 2015;8(4):393–402. 10.3233/NPM-15915043 26757008

[53] Chiruvolu A Tolia VN Qin H: Effect of delayed cord clamping on very preterm infants. *Am J Obstet Gynecol.* 2015;213(5):676.e1–7. 10.1016/j.ajog.2015.07.016 26196456

[54] Backes CH Rivera BK Haque U: Placental transfusion strategies in very preterm neonates: a systematic review and meta-analysis. *Obstet Gynecol.* 2014;124(1):47–56. 10.1097/AOG.0000000000000324 24901269

[55] Mercer JS Erickson-Owens DA Vohr BR: Effects of Placental Transfusion on Neonatal and 18 Month Outcomes in Preterm Infants: A Randomized Controlled Trial. *J Pediatr.* 2016;168:50–5.e1. 10.1016/j.jpeds.2015.09.068 26547399 PMC4698069

[56] Elimian A Goodman J Escobedo M: Immediate compared with delayed cord clamping in the preterm neonate: a randomized controlled trial. *Obstet Gynecol.* 2014;124(6):1075–9. 10.1097/AOG.0000000000000556 25415157

[57] Song D Jegatheesan P DeSandre G: Duration of Cord Clamping and Neonatal Outcomes in Very Preterm Infants. *PLoS One.* 2015;10(9):e0138829. 10.1371/journal.pone.0138829 26390401 PMC4577121

[58] Jaiswal P Upadhyay A Gothwal S: Comparison of two types of intervention to enhance placental redistribution in term infants: randomized control trial. *Eur J Pediatr.* 2015;174(9):1159–67. 10.1007/s00431-015-2511-y 25800496

[59] Rabe H Sawyer A Amess P: Neurodevelopmental Outcomes at 2 and 3.5 Years for Very Preterm Babies Enrolled in a Randomized Trial of Milking the Umbilical Cord versus Delayed Cord Clamping. *Neonatology.* 2016;109(2):113–9. 10.1159/000441891 26650133

[60] McDonald SJ Middleton P Dowswell T: Effect of timing of umbilical cord clamping of term infants on maternal and neonatal outcomes. *Evid Based Child Health.* 2014;9(2):303–97. 10.1002/ebch.1971 25404605

[61] Higgins RD Raju TN Perlman J: Hypothermia and perinatal asphyxia: executive summary of the National Institute of Child Health and Human Development workshop. *J Pediatr.* 2006;148(2):170–5. 10.1016/j.jpeds.2005.12.009 16492424

[62] Blackmon LR Stark AR: Hypothermia: a neuroprotective therapy for neonatal hypoxic-ischemic encephalopathy. *Pediatrics.* 2006;117(3):942–8. 10.1542/peds.2005-2950 16510680

[63] Gunn AJ Gunn TR: The 'pharmacology' of neuronal rescue with cerebral hypothermia. *Early Hum Dev.* 1998;53(1):19–35. 10.1016/S0378-3782(98)00033-4 10193924

[64] Drury PP Bennet L Gunn AJ: Mechanisms of hypothermic neuroprotection. *Semin Fetal Neonatal Med.* 2010;15(5):287–92. 10.1016/j.siny.2010.05.005 20646974

[65] Gunn AJ Gluckman PD Gunn TR: Selective head cooling in newborn infants after perinatal asphyxia: a safety study. *Pediatrics.* 1998;102(4 Pt 1):885–92. 9755260 10.1542/peds.102.4.885

[66] Azzopardi D Robertson NJ Cowan FM: Pilot study of treatment with whole body hypothermia for neonatal encephalopathy. *Pediatrics.* 2000;106(4):684–94. 11015509 10.1542/peds.106.4.684

[67] Gluckman PD Wyatt JS Azzopardi D: Selective head cooling with mild systemic hypothermia after neonatal encephalopathy: multicentre randomised trial. *Lancet.* 2005;365(9460):663–70. 10.1016/S0140-6736(05)17946-X 15721471

[68] Shankaran S Laptook AR Ehrenkranz RA: Whole-body hypothermia for neonates with hypoxic-ischemic encephalopathy. *N Engl J Med.* 2005;353(15):1574–84. 10.1056/NEJMcps050929 16221780

[69] Azzopardi DV Strohm B Edwards AD: Moderate hypothermia to treat perinatal asphyxial encephalopathy. *N Engl J Med.* 2009;361(14):1349–58. 10.1056/NEJMoa0900854 19797281

[70] Zhou WH Cheng GQ Shao XM: Selective head cooling with mild systemic hypothermia after neonatal hypoxic-ischemic encephalopathy: a multicenter randomized controlled trial in China. *J Pediatr.* 2010;157(3):367–72, 372.e1–3. 10.1016/j.jpeds.2010.03.030 20488453

[71] Simbruner G Mittal RA Rohlmann F: Systemic hypothermia after neonatal encephalopathy: outcomes of neo.nEURO.network RCT. *Pediatrics.* 2010;126(4):e771–8. 10.1542/peds.2009-2441 20855387

[72] Jacobs SE Morley CJ Inder TE: Whole-body hypothermia for term and near-term newborns with hypoxic-ischemic encephalopathy: a randomized controlled trial. *Arch Pediatr Adolesc Med.* 2011;165(8):692–700. 10.1001/archpediatrics.2011.43 21464374

[73] Orbach SA Bonifacio SL Kuzniewicz MW: Lower incidence of seizure among neonates treated with therapeutic hypothermia. *J Child Neurol.* 2014;29(11):1502–7. 10.1177/0883073813507978 24334344 PMC4053513

[74] Rutherford M Ramenghi LA Edwards AD: Assessment of brain tissue injury after moderate hypothermia in neonates with hypoxic-ischaemic encephalopathy: a nested substudy of a randomised controlled trial. *Lancet Neurol.* 2010;9(1):39–45. 10.1016/S1474-4422(09)70295-9 19896902 PMC2795146

[75] Jacobs SE Berg M Hunt R: Cooling for newborns with hypoxic ischaemic encephalopathy. *Cochrane Database Syst Rev.* 2013; (1):CD003311. 10.1002/14651858.CD003311.pub3 23440789 PMC7003568

[76] Gano D Chau V Poskitt KJ: Evolution of pattern of injury and quantitative MRI on days 1 and 3 in term newborns with hypoxic-ischemic encephalopathy. *Pediatr Res.* 2013;74(1):82–7. 10.1038/pr.2013.69 23618911

[77] Bonifacio SL Saporta A Glass HC: Therapeutic hypothermia for neonatal encephalopathy results in improved microstructure and metabolism in the deep gray nuclei. *AJNR Am J Neuroradiol.* 2012;33(11):2050–5. 10.3174/ajnr.A3117 22595900 PMC3473161

[78] Shankaran S Pappas A McDonald SA: Childhood outcomes after hypothermia for neonatal encephalopathy. *N Engl J Med.* 2012;366(22):2085–92. 10.1056/NEJMoa1112066 22646631 PMC3459579

[79] Guillet R Edwards AD Thoresen M: Seven- to eight-year follow-up of the CoolCap trial of head cooling for neonatal encephalopathy. *Pediatr Res.* 2012;71(2):205–9. 10.1038/pr.2011.30 22258133

[80] Azzopardi D Strohm B Marlow N: Effects of hypothermia for perinatal asphyxia on childhood outcomes. *N Engl J Med.* 2014;371(2):140–9. 10.1056/NEJMoa1315788 25006720

[81] Shankaran S Laptook AR Pappas A: Effect of depth and duration of cooling on deaths in the NICU among neonates with hypoxic ischemic encephalopathy: a randomized clinical trial. *JAMA.* 2014;312(24):2629–39. 10.1001/jama.2014.16058 25536254 PMC4335311

[82] Gonzalez FF Larpthaveesarp A McQuillen P: Erythropoietin increases neurogenesis and oligodendrogliosis of subventricular zone precursor cells after neonatal stroke. *Stroke.* 2013;44(3):753–8. 10.1161/STROKEAHA.111.000104 23391775 PMC3689426

[83] Dixon BJ Reis C Ho WM: Neuroprotective Strategies after Neonatal Hypoxic Ischemic Encephalopathy. *Int J Mol Sci.* 2015;16(9):22368–401. 10.3390/ijms160922368 26389893 PMC4613313

[84] Wu YW Bauer LA Ballard RA: Erythropoietin for neuroprotection in neonatal encephalopathy: safety and pharmacokinetics. *Pediatrics.* 2012;130(4):683–91. 10.1542/peds.2012-0498 23008465 PMC3457622

[85] Wu YW Mathur AM Chang T: High-Dose Erythropoietin and Hypothermia for Hypoxic-Ischemic Encephalopathy: A Phase II Trial. *Pediatrics.* 2016;137(6): pii: e20160191. 10.1542/peds.2016-0191 27244862

[86] Azzopardi D Robertson NJ Bainbridge A: Moderate hypothermia within 6 h of birth plus inhaled xenon versus moderate hypothermia alone after birth asphyxia (TOBY-Xe): a proof-of-concept, open-label, randomised controlled trial. *Lancet Neurol.* 2015;15(2):145–153, pii: S1474-4422(15)00347-6. 10.1016/S1474-4422(15)00347-6 26708675 PMC4710577

[87] Novak I Walker K Hunt RW: Concise Review: Stem Cell Interventions for People With Cerebral Palsy: Systematic Review With Meta-Analysis. *Stem Cells Transl Med.* 2016;5(8):1014–25. 10.5966/sctm.2015-0372 27245364 PMC4954458

